# SiC resistive X-ray beam monitor for intensity and position control of synchrotron light

**DOI:** 10.1107/S1600577526005242

**Published:** 2026-06-18

**Authors:** Gabriele Trovato, Niccolò La Rosa, Francesco La Via, Janin Lubeck, Antonio Manno, Samuele Moscato, Matthias Müller, Massimo Camarda

**Affiliations:** a Università degli Studi di Catania Dipartimento di Fisica e Astronomia ‘Ettore Majorana’ Via Santa Sofia 64 95123Catania Italy; b STLab srl, Via Anapo 53, 95126Catania, Italy; c Istituto Nazionale di Fisica Nucleare – INFN Sezione di Catania Via S. Sofia 64 95123Catania Italy; d Istituto per la Microelettronica e Microsistemi CNR-IMM Sezione di Catania, Strada VIII Zona Industriale 5 95121Catania Italy; e Università degli Studi di Catania, Dipartimento di Ingegneria Elettrica, Elettronica e Informatica (DIEEI), Viale Andrea Doria 6, I-95125Catania, Italy; f Physikalisch-Technische Bundesanstalt (PTB) Abbestraße 2-12 10587Berlin Germany; g SenSiC GmbH, DeliveryLAB, 5234Villigen, Switzerland; European XFEL, Germany

**Keywords:** silicon carbide, free-standing membrane, beamline diagnostic element, X-ray position monitoring, lateral effect photodiode (LEP)

## Abstract

A silicon carbide (SiC) X-ray beam position monitor is presented, based on a resistive charge-division principle derived from lateral-effect photodiodes and specifically adapted for synchrotron radiation applications. This device, referred to as a resistive X-ray beam position monitor (rXBPM), exploits a free-standing SiC membrane combined with a resistive p^+^-doped layer, enabling transmission-mode operation while preserving high radiation hardness and mechanical robustness. In contrast to conventional segmented X-ray beam position monitors, whose response depends strongly on the beam spot size and is typically limited to narrow linear regions, the resistive architecture of the rXBPM provides an intrinsically beam-footprint-independent position signal with an extended linear response region. The detector was fabricated using selective electrochemical etching to realize a thin membrane structure and was experimentally characterized at the microfocus beamline (MiFo) in the PTB laboratory at the BESSY II synchrotron facility using 5.4 keV X-rays. An average transmission of approximately 61% was measured, with good spatial uniformity across the membrane area. Raster-scan measurements demonstrate a linear position response over ranges of ±500 µm and ±1 mm around the detector center, with position sensitivities exceeding 0.157 mm^−1^ and estimated upper-limit noise-equivalent positions of a few micrometres. Three-dimensional COMSOL simulations were used to model charge transport and lateral charge division in the real device geometry, showing excellent agreement with experimental results and confirming the independence of the position sensitivity from the beam spot size over a wide range of operating conditions. These results establish SiC rXBPMs as a compact, beam spot size calibration-free and radiation-hard solution for beam diagnostics at modern synchrotron light sources, with particular relevance for applications requiring large active areas, extended linearity and minimal beam perturbation.

## Abstract 2

The characterization of a silicon carbide free-standing membrane resistive detector for spot-size-independent X-ray beam position monitoring in transmission is presented.

## 1. Introduction

One of the requirements of modern synchrotron radiation beamlines is the continuous monitoring of X-ray beam parameters, because the stability of X-ray beams directly influences the reproducibility of experiments and the safe operation of beamline optics. In a detailed review by Bilderback *et al.* (2005 ▸), beam diagnostics are established as a staple of synchrotron facilities, allowing the management of a series of beam parameters under the rigorously growing conditions of experiments. In particular, the precise definition of the position of the photon beam is a requirement, because a small drift of the position generates misalignment and a change of the number of incoming photons for the experiment station.

Moving to higher brilliance light sources has required increasingly tight criteria for beam position stability, thus reaching the sub-micrometric spatial resolution. This has been shown in specific scientific efforts on high-precision beam positioning monitoring, in which fast and precise methods are necessary for monitoring the beam movement in real time (Bunk *et al.*, 2005 ▸). This necessity has been further increased by the advent of the fourth-generation synchrotrons and advanced methods in experiments, such as macromolecular crystallography, in which the stable delivery of beams is important to maximize the use of state-of-the-art instruments (Owen *et al.*, 2016 ▸).

A successful XBPM for current beamlines should, therefore, offer real-time position data and should work in a minimally invasive way, while being fully compatible with high photon flux, granting stable operations. Solid-state devices made out of wide-bandgap semiconducting material are currently the best candidates for satisfying the needs of modern-generation synchrotron light sources. Single-crystal diamond grown by CVD (scCVD) technology is considered a reference for beam monitoring because of its high radiation resistance, speed, and thermal conductivity. Diamond-based X-ray beam position monitors (XBPMs) using segmented electrodes have been widely studied and proven for online beam position monitoring (Bergonzo *et al.*, 1999 ▸; Desjardins *et al.*, 2013 ▸; Griesmayer *et al.*, 2016 ▸). One of the materials recently gaining interest is silicon carbide (SiC), which offers high radiation resistance along with the potential to create thin, sturdy free-standing membranes thanks to the doping selective electrochemical etching (ECE) technique. Experimental assessment of SiC-based XBPMs was conducted by Nida *et al.* (2019 ▸), proving that SiC can achieve transparency similar to that of diamond detectors with equal signal linearity and about twice the signal-to-noise ratio when exposed to the synchrotron beam, considering the energy needed to form an electron–hole pair in the two materials [*E*_diamond_ = 13.8 eV (Kraus *et al.*, 2021 ▸) versus *E*_SiC_ = 7.6 eV (De Napoli, 2022 ▸)]. A direct comparison between an scCVD and SiC free-standing membrane has been performed by Houghton *et al.* (2023 ▸), finding SiC as a diamond equivalent in operative performances, with the advantage of having a larger area granting the possibility to perform alignment measurements even for large beam sizes. Due to the presence of silicon atoms, SiC exhibits a higher photoelectric absorption coefficient than diamond at equal thickness, reaching up to about one order of magnitude in the tender X-ray range. To address this problem, SiC membranes working as X-ray beam intensity and position monitors (XBIM and XBPM), with thickness less than 2 µm, were recently fabricated and validated (Trovato *et al.*, 2025*a* ▸; Trovato *et al.*, 2025*b* ▸), and further thinned down to approximately 200 nm (Medina *et al.*, 2025 ▸; Trovato *et al.*, 2025*c* ▸).

However, conventional four-quadrant photodiodes suffer from spot-size dependence, strongly limiting their adaptability to different beam conditions. In Medina *et al.* (2025 ▸), a strong relation between the interpad distance, the device thickness and the beam’s full width at half-maximum (FWHM) is accurately described as a limiting factor for this type of detector. To overcome this issue, the best detector structure could be the lateral-effect photodiodes (LEPs), which are able to locate the centroid of the incident beam independent of the light spot size and shape (Mäkynen, 2000 ▸). LEPs are continuous and large-area detectors with the contacts placed on the edges of the devices. The working mechanism is based on the charge division on a resistive sheet on top of the active area of the detector. The incident X-ray beam creates electron–hole pairs within the illuminated area. This photocurrent is re-distributed through the resistive p^+^-doped layer under the influence of the boundary conditions set by the geometry of contacts. The current collected by each contact will depend on the position of the beam on the active surface. For large beam spot size, the position of the beam will actually be its centroid. LEPs can come in one-axis and two-axis configurations and with different contact displacements and geometries. The most relevant are the Wallmark, the duolateral, the tetralateral and the pin-cushion configuration, each with different features in terms of linearity, position resolution, and thermal noise effect. Extensive work on these devices has been performed with silicon technology and reported by Woltring (1975 ▸), Noorlag (1982 ▸), Solal *et al.* (2002 ▸) and Cowin & Watson (1997 ▸).

In the case of synchrotron facilities, silicon detectors are not suitable for high-brilliance beams. Diamond-based duo-lateral X-ray beam position monitors have been demonstrated by Pomorski *et al.* (2009 ▸) as a homogeneous 4 mm × 4 mm scCVD diamond detector with diamond-like-carbon resistive electrodes covering a 2.93 mm × 2.93 mm active area. This type of device is optimized for different applications, from soft X-rays beamlines, such as Sirius at the SOLEIL synchrotron facility (Desjardins *et al.*, 2014 ▸), to extreme conditions, like in the EuXFEL (Çonka Yıldız *et al.*, 2024 ▸).

Recent advances in SiC technology have now made it possible to fabricate an SiC tetralateral position-sensitive detector (PSD) (Wang *et al.*, 2024 ▸). The device demonstrated very low dark current at zero bias, high UV responsivity, fast response, and reliable two-dimensional position sensing with limited nonlinearity on 76% of the active area. As already mentioned, the position response was demonstrated to be independent of the incident light power, spot size, and applied bias voltage. Although this work focused on UV optical characterization on a bulk sensor, it provides a clear validation of SiC-based lateral-effect PSDs as mature and reliable position-sensitive devices. From this perspective, SiC lateral-effect PSDs represent a highly promising platform for X-ray beam position monitoring (XBPM) with an active sensing area up to four times that of current diamond-based XBPMs.

In this paper, we report on the design, realization, and characterization of the first silicon carbide lateral-effect PSD with a free-standing membrane structure specifically developed for X-ray beam position monitoring (rXBPM). The present study explores the possibility of producing a lateral-effect photodiode featuring a tetralateral geometry working in transmission thanks to the free-standing membrane, suitable for the beamline requirements, and extends the application of SiC PSDs to the X-ray regime, where mechanisms of charge generation, carrier transport conditions, and operational constraints are different from those encountered in optical measurements. This work intends to provide proof-of-principle that SiC lateral-effect PSDs are a viable and competitive solid-state solution for rXBPMs, providing a compact, radiation-tolerant alternative to existing beam position monitoring technologies.

## 2. Materials and methods

### 2.1. Device fabrication

**Figure 1:**
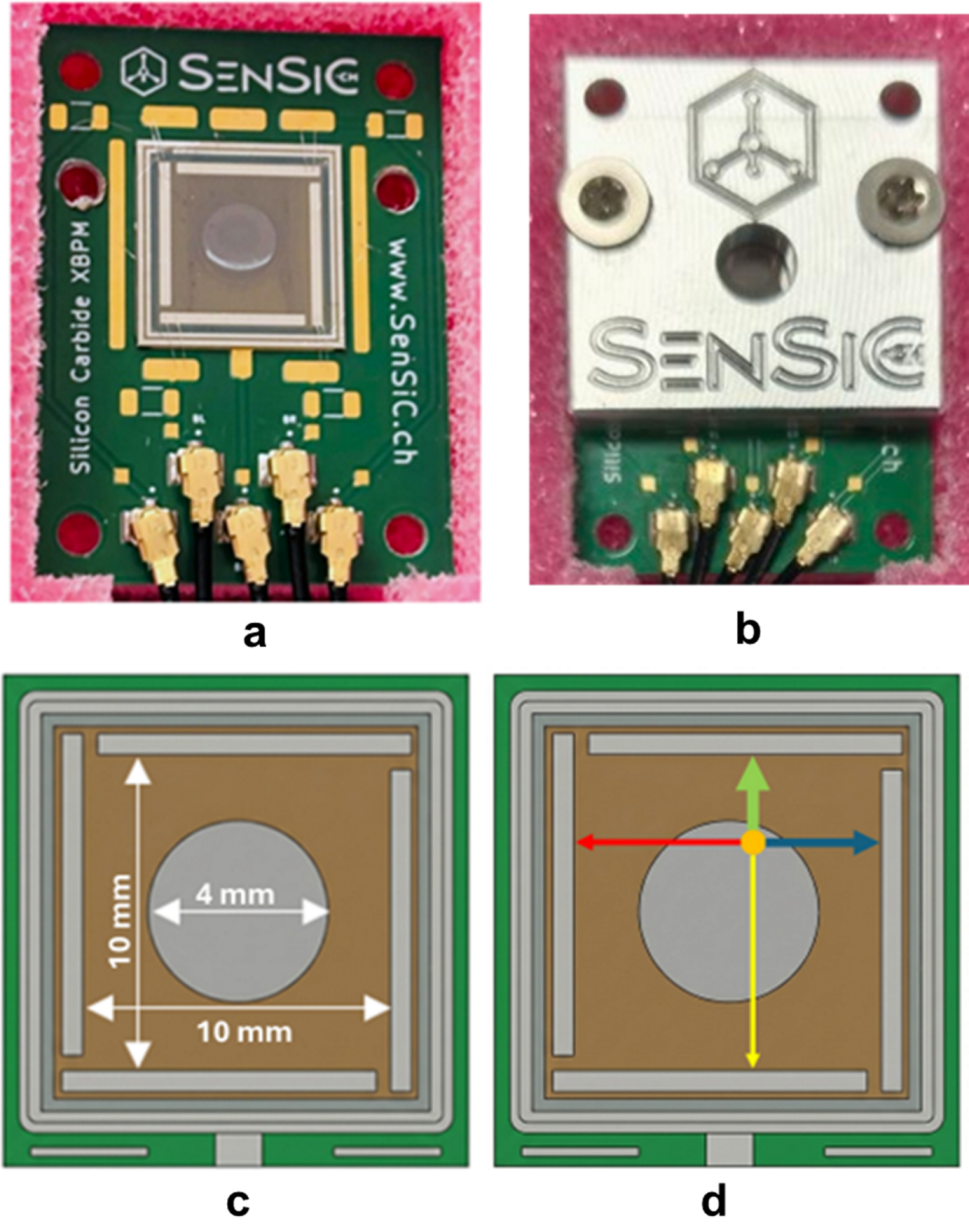
(*a*) Image of the SiC rXBPM and (*b*) of the protective cover of the device. (*c*) Schematic sketch of the rXBPM with its dimensions. (*d*) Schematic representation of the charge splitting among the four channels. The arrow is thicker the nearer the incidence point of the beam is to the metallic contact.

Different 4H-SiC rXBPMs were fabricated by SenSiC GmbH, with three nominal thicknesses of the n^−^-doped active layer available: 1 µm, 10 µm and 34 µm. An image of the 10 µm device is represented in Fig. 1 ▸ The device consists of a 370 µm SiC n^+^-doped (10^18^ cm^−3^) substrate, n^−^-doped (5 × 10^14^ cm^−3^) active layer and, a 0.3 µm p^+^-doped (10^19^ cm^−3^) SiC. The n-layer doping is given by nitrogen atoms included in the growth process, while the p-doping is realized by aluminium ion implantation. The p-doped layer is the resistive layer, which allows the device to function as an LEP. The measured sheet resistance associated with this layer is 52 kΩ □^−1^, in the range required for lateral-effect operation.

On the four sides of the detector, 10 mm apart in both directions, four 1 mm × 12 mm Ohmic metallic contacts are realized, using 80 nm of Ti and 300 nm of Al, combining the physical vapor deposition technique and a rapid thermal annealing process. The disposition of the four electrodes, as shown in Fig. 1 ▸, is adequately chosen to realize a tetralateral configuration device. Additional metallic stacks of Ti and Al, for a total thickness of 2.3 µm, are deposited to grant low series resistances and facilitate the wire bonding process.

On the back of the 1 µm and 10 µm devices, an ECE procedure is performed, similar to that reported by Nida *et al.* (2019 ▸), to carve a 4 mm-diameter hole on the n^+^ substrate. This process creates what is called a ‘free-standing membrane’, which thins the device down to make it suitable for synchrotron applications. A 50 nm Al layer is then deposited on the back of the detector to ensure electrical connection through a Schottky contact.

In this work, the characterization of the 10 µm free-standing membrane device at a synchrotron facility is presented. Additional measurements on a 10 µm device from the same batch were conducted in an open-air setup with a 5.4 keV XOS X-beam X-ray tube laboratory source (XOS, 2026 ▸). Preliminary tests on a 1 µm membrane and a 34 µm bulk device were also performed and are presented as supporting information.

### 2.2. Experimental setup

#### 2.2.1. Beam time at the MiFo beamline

**Figure 2:**
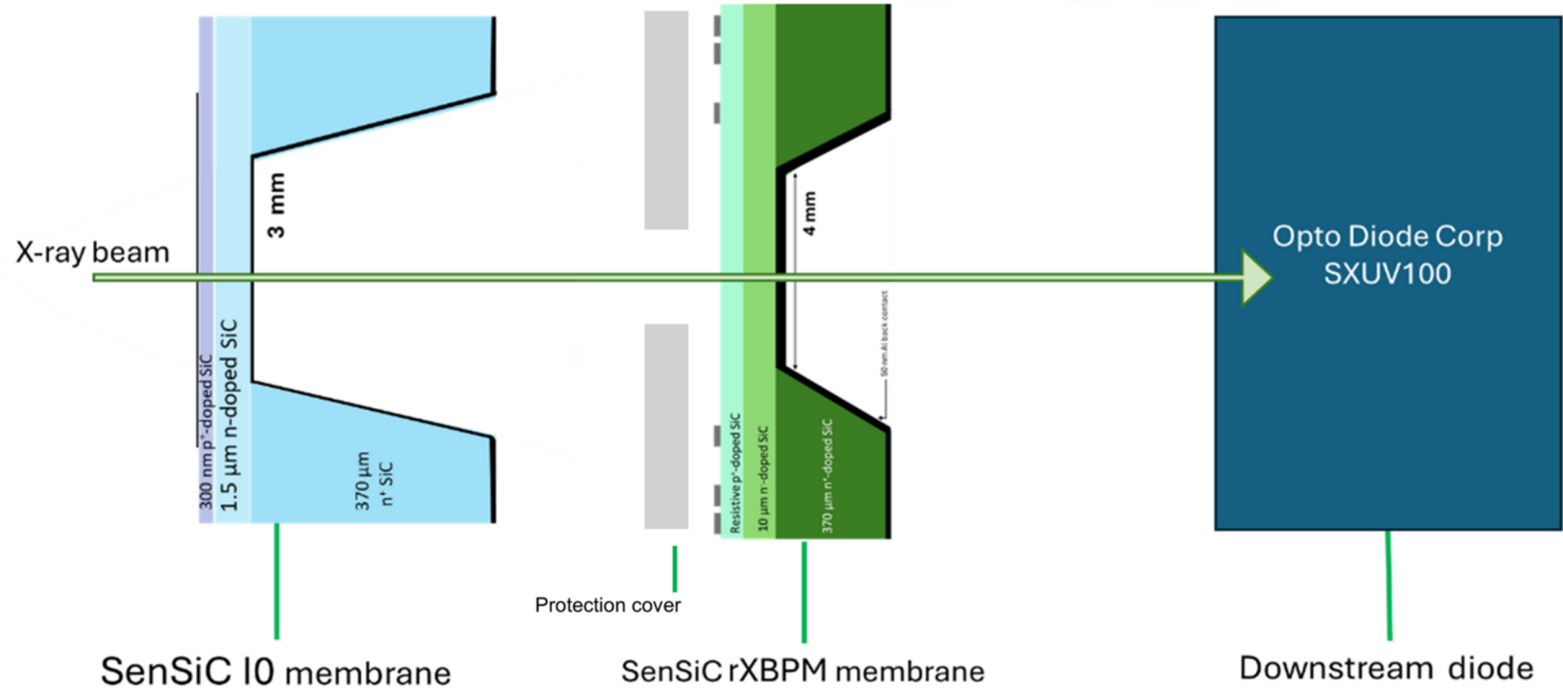
Schematic representation of the setup used at the MiFo beamline in the PTB laboratory at the BESSY II synchrotron radiation facility.

The characterization of this device has been performed at the microfocus (MiFo) beamline (Müller *et al.*, 2025 ▸) of the Physikalisch-Technische Bundesanstalt laboratory, at the BESSY II synchrotron facility. A schematic representation of the setup used for this measurement is presented in Fig. 2 ▸.

X-rays of energy 5.4 keV, with an average flux of 7.4 × 10^10^ s^−1^ and an estimated beam spot size of 25 µm, were utilized. The device was shielded by a metallic protection cover to avoid crushing the wire bondings during the mounting stage and placed inside a vacuum chamber on a motorized stage, accompanied by an Opto Diode Corp SXUV100, positioned downstream relative to the beam direction. Upstream of these detectors, a thin (1.5 µm) SenSiC SiC XBIM diode, fully characterized and reported by Trovato *et al.* (2025*a* ▸), was used as a monitor detector. The current value of the four channels of the rXBPM were measured with no bias applied, using a SenSiC PCR4 low-noise picoammeter specifically designed for the simultaneous amplification and measurement of currents from four independent channels (SenSiC, 2024 ▸).

#### 2.2.2. Test with the XOS laboratory source

Laboratory measurements have been made in air with a 5.4 keV XOS X-beam X-ray tube source (XOS, 2026 ▸) applied to 10 µm-thick SiC rXBPM manufactured in the same batch as the detector tested at the MiFo beamline.

The measurements of bias dependence have been made using the 30 kV, 1 mA XOS X-beam X-ray tube source. Beam-induced current has been determined as a sum of electrode currents, while leakage current has been recorded at identical bias conditions without applying X-rays. Measurements in reverse-bias mode up to 60 V have been made with the use of a Keithley 2410 sourcemeter (Keithley Instruments, 1998 ▸), while up to 20 V an additional measurement has been made with the SenSiC PCR4 picoammeter.

More tests have been carried out at 0 V using the PCR4 in an effort to replicate the unbiased conditions under which the synchrotron tests were carried out. A line scan was performed by moving the detector in the *y*-direction at position *x* = 0 to evaluate the uniformity of the summed current in the membrane region and establish whether there was any reduction in the current recorded during the synchrotron test as being specific to the device. Moreover, the temporal response of the detector was assessed through recording the total current as the X-ray source shutter was opened and closed.

### 2.3. Device simulation

A preliminary 2D simulation of the SiC rXBPM device, realized using a TCAD Sentaurus, has been reported by Medina *et al.* (2025 ▸). The results extracted from this simulation show that a doping concentration threshold of 10^16^ cm^−3^ is needed by the resistive layer to achieve linearity in beam position determination and no charge collection efficiency degradation at the device center. However, TCAD simulations do not permit the study of real size devices on the scale of mm^2^.

For this reason, the software package COMSOL *Multiphysics* (https://www.comsol.com) was used to model the electrostatic and charge transport properties of the detector. With this approach we could simulate the geometry of the membrane area of the real device, considering the electrodes at their real positions.

Simplified two-dimensional models were employed to qualitatively compare the lateral response of a conventional segmented XBPM and of a resistive XBPM. The models presented in the supporting information illustrate how the position sensitivity of standard XBPMs depend on the beam spot size. The 2D model realized in COMSOL was not able to fully reproduce the non-linear behavior at the sides of the tetralateral LEP. For this reason, the 3D simulation of the device was performed to investigate the detector’s spatial response. In this case, the simulation was not performed using a full drift–diffusion semiconductor model, as such an approach is computationally impractical for a device of this size. The detector response was investigated using the standard *Electric Currents* module in COMSOL *Multiphysics*, which provides a simplified electrostatic conduction description of the system. The active area of the detector was modeled, from bottom to top, as: (i) a thicker low-conductivity layer representing the n^−^-doped SiC region, (ii) a thinner higher-conductivity layer representing the p^+^-doped SiC resistive layer, and (iii) four lateral terminals placed at the edges of the top surface, representing the front-side electrical contacts. The conductivity of the resistive layer was set to 64 S m^−1^, a value obtained from the measured sheet resistance of 52 kΩ □^−1^ and the layer thickness of 0.3 µm.

The FWHM of the beam has been spanned from 1 µm up to 2 mm to confirm, even with the three-dimensional model, the beam spot size independence of this type of device. Between the front and the back of the device, a 0.5 V bias is applied. The small bias used in the simplified *Electric Currents* model was introduced only to define the vertical conduction path and does not affect the lateral charge-division mechanism discussed here.

A parametric sweep was performed on the parameter *x*, representing the position of the charge generation area along the axis of the device surface. This procedure effectively reproduces a line scan, similar to moving a focused beam across the sensor in an experiment, and provides insight into the spatial uniformity and symmetry of the detector response.

To ensure numerical stability and preserve spatial resolution, an adaptive mesh was adopted. In particular, a cylindrical mesh domain of the size of the beam FWHM was introduced in both layers around the charge-generation region. Everywhere else, a less dense mesh was used farther away from the irradiated area. To ensure convergence of the 3D COMSOL simulations without introducing excessive model complexity, the membrane thickness was increased by a factor of four with respect to the experimental device. This modification does not affect the qualitative lateral charge-division behavior discussed in this work.

## 3. Results and discussion

### 3.1. Characterization at the MiFo beamline

The transmittance map of the SiC free-standing membrane is obtained, using 5.4 keV X-rays, by measuring the current produced by the upstream and downstream diodes, and performing the measurement with and without the SiC device in between the two of them. The ratio between the two currents when there is no membrane in the middle gives the background reference measure (*r*_bg_). The ratio of the two currents when the SiC membrane is inserted, divided by *r*_bg_, is the transmittance of the entire exposed area. The raster scan has been performed using a spot size of 25 µm × 25 µm and a step of 150 µm in both *x* and *y*.

**Figure 3:**
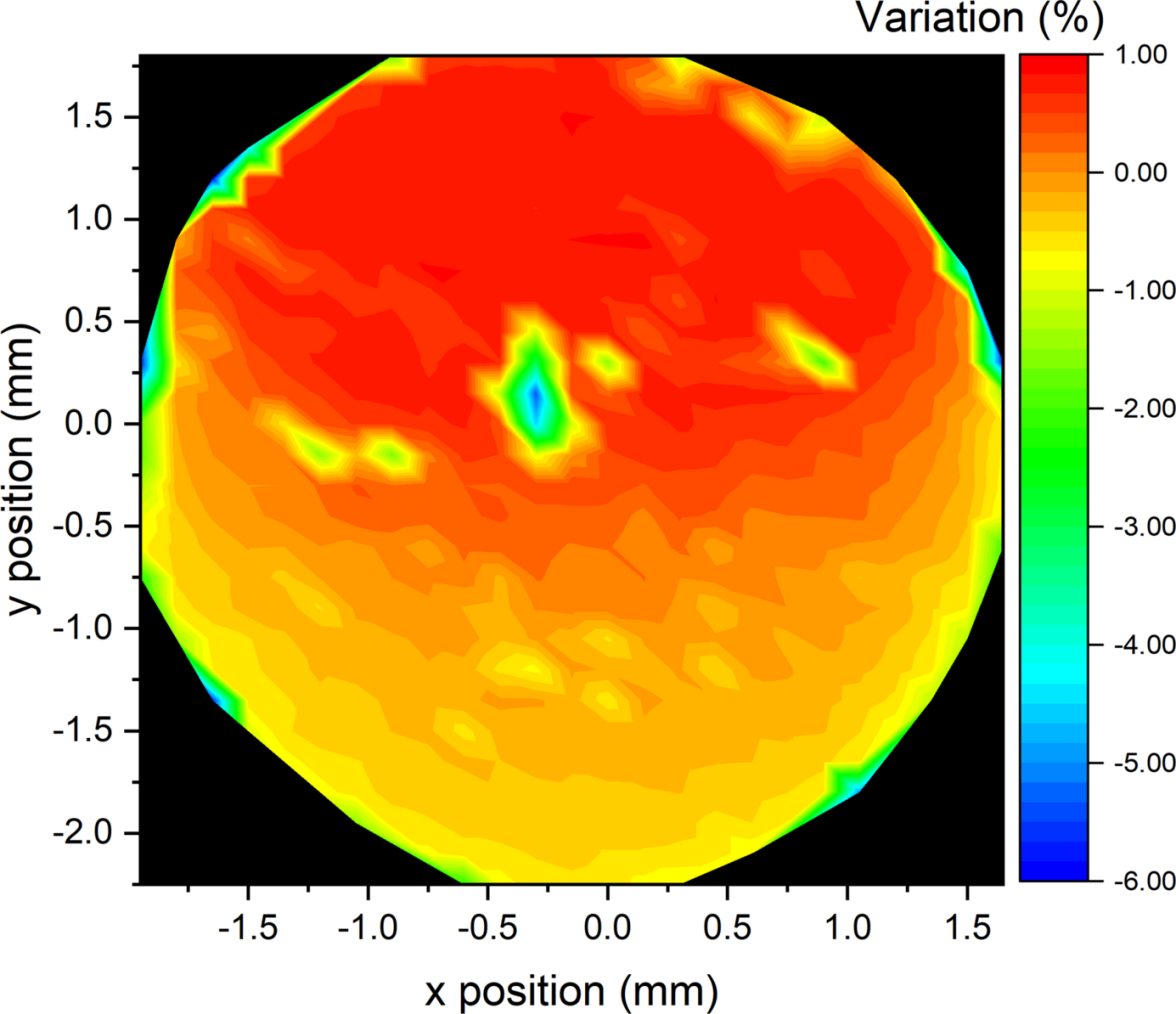
Map of transmission percentage variation of the SiC rXBPM membrane performed using 5.4 keV X-rays. The variation is calculated with respect to the average transmittance coefficient of 0.606

The average transmittance coefficient of the membrane is 0.606, with a standard deviation of 0.005. The transmission map of Fig. 3 ▸ shows the percentage variation of transmission with respect to the average value. The membrane shows one region with reduced transmission, probably a measurement artifact since there is no related effect in the electrical performances, and a vertical trend of ∼1% difference in transmission going from the bottom to the top of the membrane. Despite these features, the membrane shows an overall good uniformity of the etched surface.

**Figure 4:**
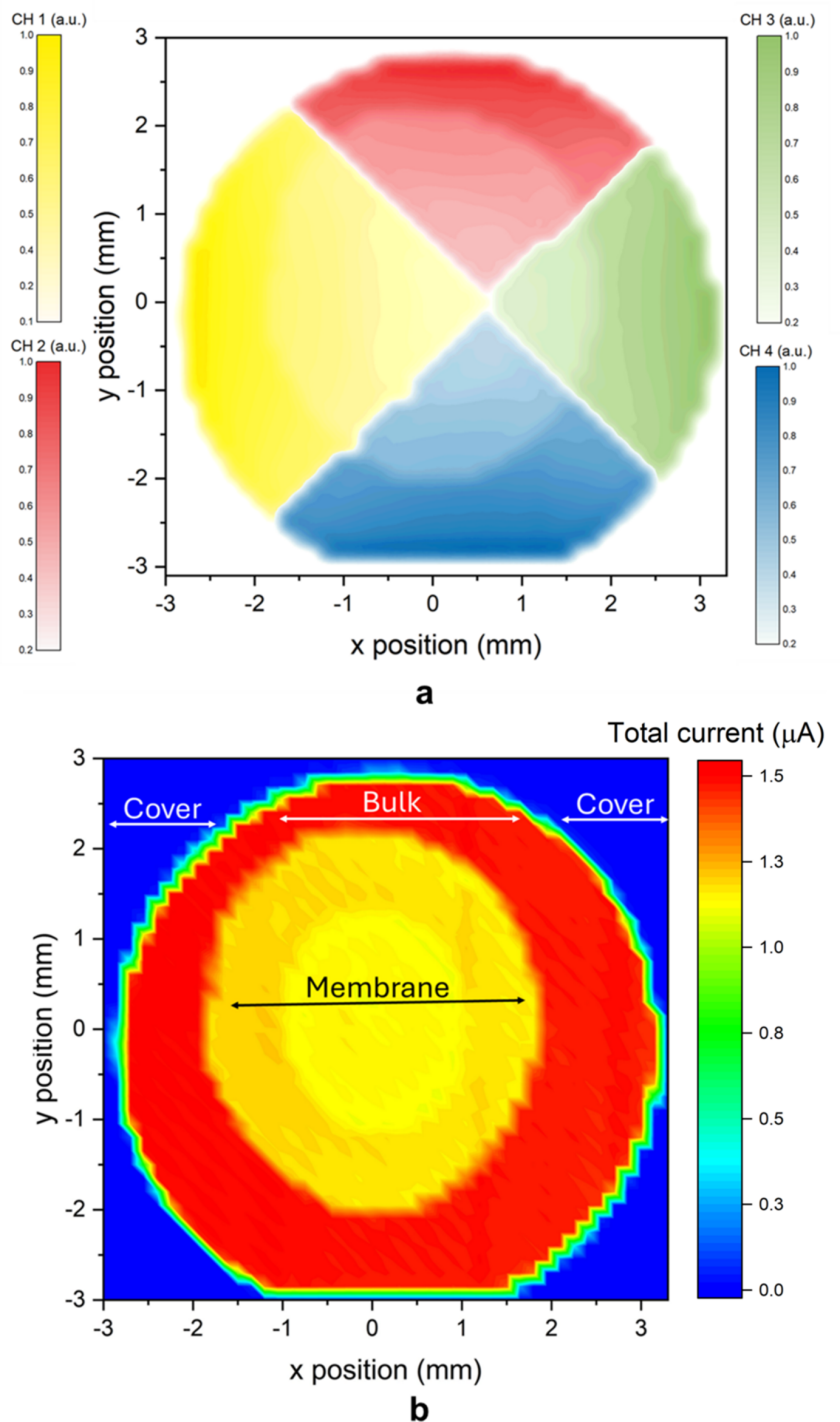
(*a*) Current collected by the single channels during the raster scan measurement. (*b*) Sum of the currents measured by the four channels during the irradiation.

**Figure 5:**
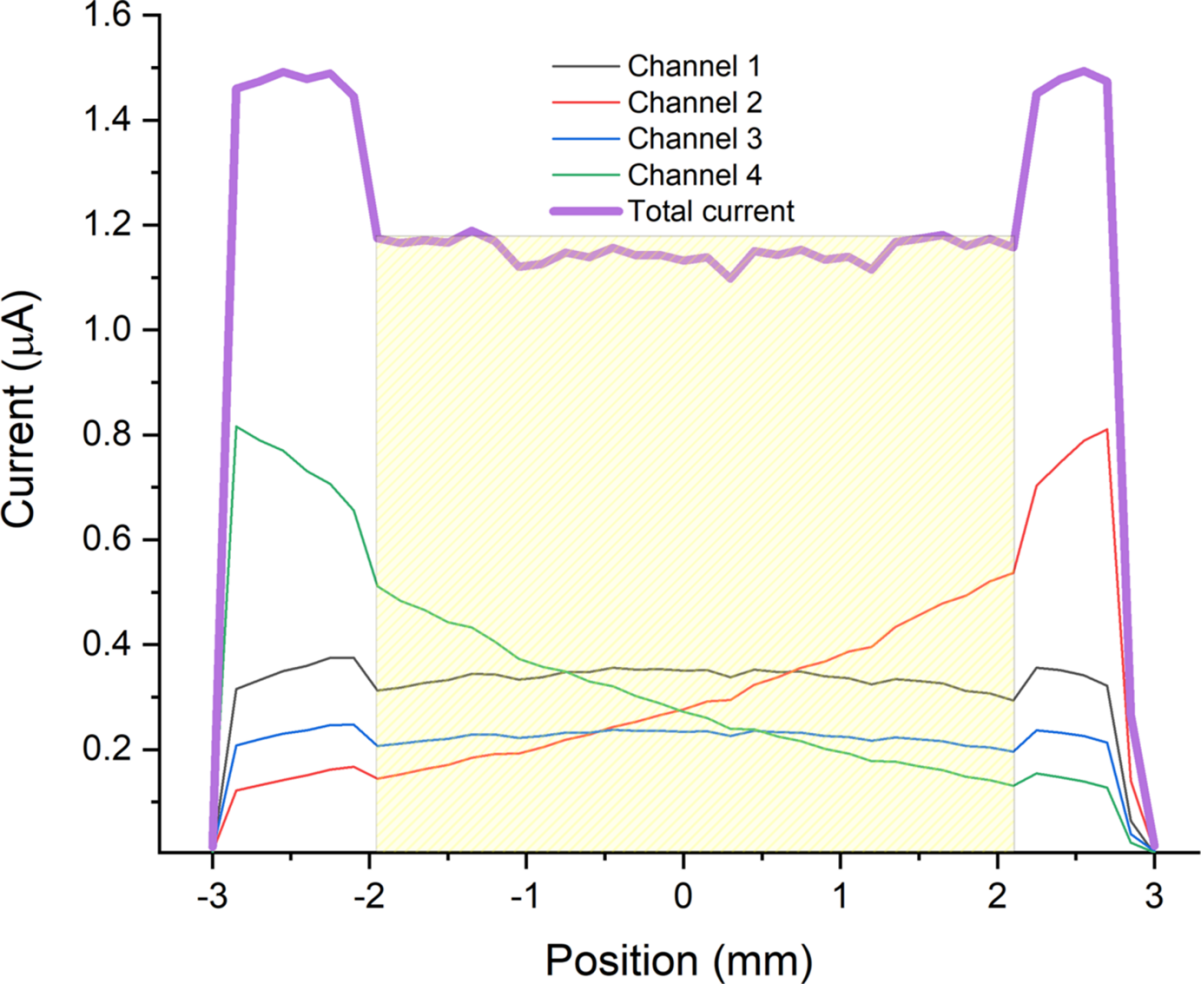
Current collected by the four channels and sum of the currents measured during a line scan irradiation. The yellow region indicates the position of the etched free-standing membrane.

From the same raster scan, done using 5.4 keV photons, with an average flux of 7.4 × 10^10^ s^−1^, the signal response of the device is estimated along the entire exposed area. Fig. 4 ▸(*a*) shows the normalized beam-induced current collected by the four electrodes during the raster scan, consistent with COMSOL’s results. The current measured by one channel tends to be higher the nearer the beam is to the corresponding electrode and to split accordingly to the resistive path between the incidence spot and the electrode. The current produced by the membrane area, being lower in value, is represented by lighter colors compared with the surrounding bulk region. The sum of the currents read by the four channels is represented in Fig. 4 ▸(*b*). The red region corresponds to the region of the device not carved through electrochemical etching. In this area, a contribution to the signal is given by the charge carriers generated in the n^+^-doped layer, as reported by Trovato *et al.* (2025*a* ▸). Within the yellow area, the signal collected comes from the free-standing membrane. The average current sum measured in the membrane area is 1.16 µA, with a standard deviation of 0.02 µA. A line scan is extracted from Fig. 4 ▸(*b*) at the position *x* = 0 mm and shown in Fig. 5 ▸ to highlight the symmetric effect of current separation among the four metallic contacts. The yellow area of the plot represents the membrane region of the device. Outside the yellow area, Fig. 5 ▸ shows the current of the unetched region of the device. A comparison between the simulated and the experimental results is shown later in Fig. 11(*a*).

**Figure 6:**
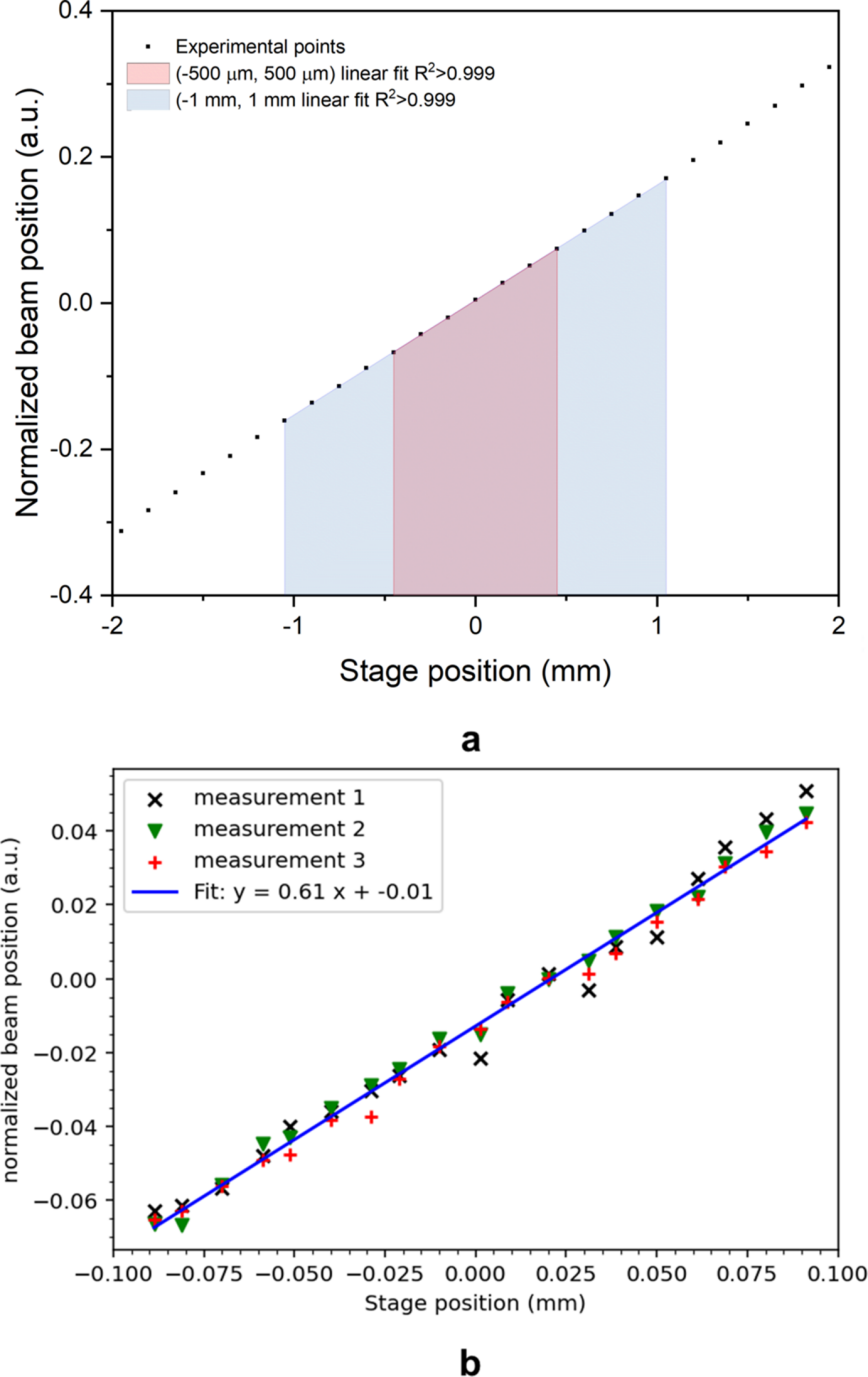
(*a*) Normalized beam position as a function of the motorized stage displacement measured with the SiC resistive X-ray beam position monitor. Black squares represent experimental data points. The shaded regions indicate the intervals used for linear regression: ±500 µm (red) and ±1 mm (blue). (*b*) Short-range measurements (±100 µm) of the normalized beam position with respect to the stage position.

The spatial sensitivity of the XBPM was extracted from the line-scan data by calculating the beam position measured by the device as follows, 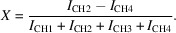
Performing a linear fit of the position measured by the device (*X*) versus the displacement of the motorized stage (*x*) [Fig. 6 ▸(*a*)], an excellent linear response (*R*^2^ > 0.999) within ±500 µm and ±1 mm around the center is found, with a lateral sensitivity (*dX*/*dx*) of 0.1570 ± 0.0006 mm^−1^ and 0.1573 ± 0.0003 mm^−1^, respectively. Due to the lack of a dedicated fixed-position acquisition, the noise-equivalent position (NEP) was estimated as NEP = 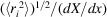, with *r*_*i*_ the residuals of the linear fit in the two regions. Using the RMS value of the residuals within (σ_500µm_ = 0.0004 and σ_1mm_ = 0.0007), NEPs of approximately 2.7 µm and 4.7 µm were estimated. These values represent an upper limit to the position resolution of the rXBPM.

Three additional short-range measurements [Fig. 6 ▸(*b*)] were performed in the ±100 µm region using a 150 µm beam spot size to assess the repeatability of beam position determination. The measurement was performed using four Keysight 2985 electrometers. From a linear fit of the normalized position, a local lateral sensitivity of 0.615 ± 0.008 mm^−1^ was extracted. The RMS of the fit residuals was used to estimate the single-point position precision, resulting in a value of approximately 5 µm.

The obtained position noise level should be viewed as a conservative maximum upper bound of the resolution of this detector, as no dedicated measurement has been performed. Optimized diamond XBPMs and 4H-SiC XBPMs show a sub-micrometre resolution with a position noise level of approximately 100 nm at FMX (Schneider *et al.*, 2021 ▸), in duo-lateral diamond detectors at SOLEIL (Desjardins *et al.*, 2018 ▸), and upper limit of position noise level of 187 nm for SiC membranes at DLS (Houghton *et al.*, 2023 ▸).

**Figure 7:**
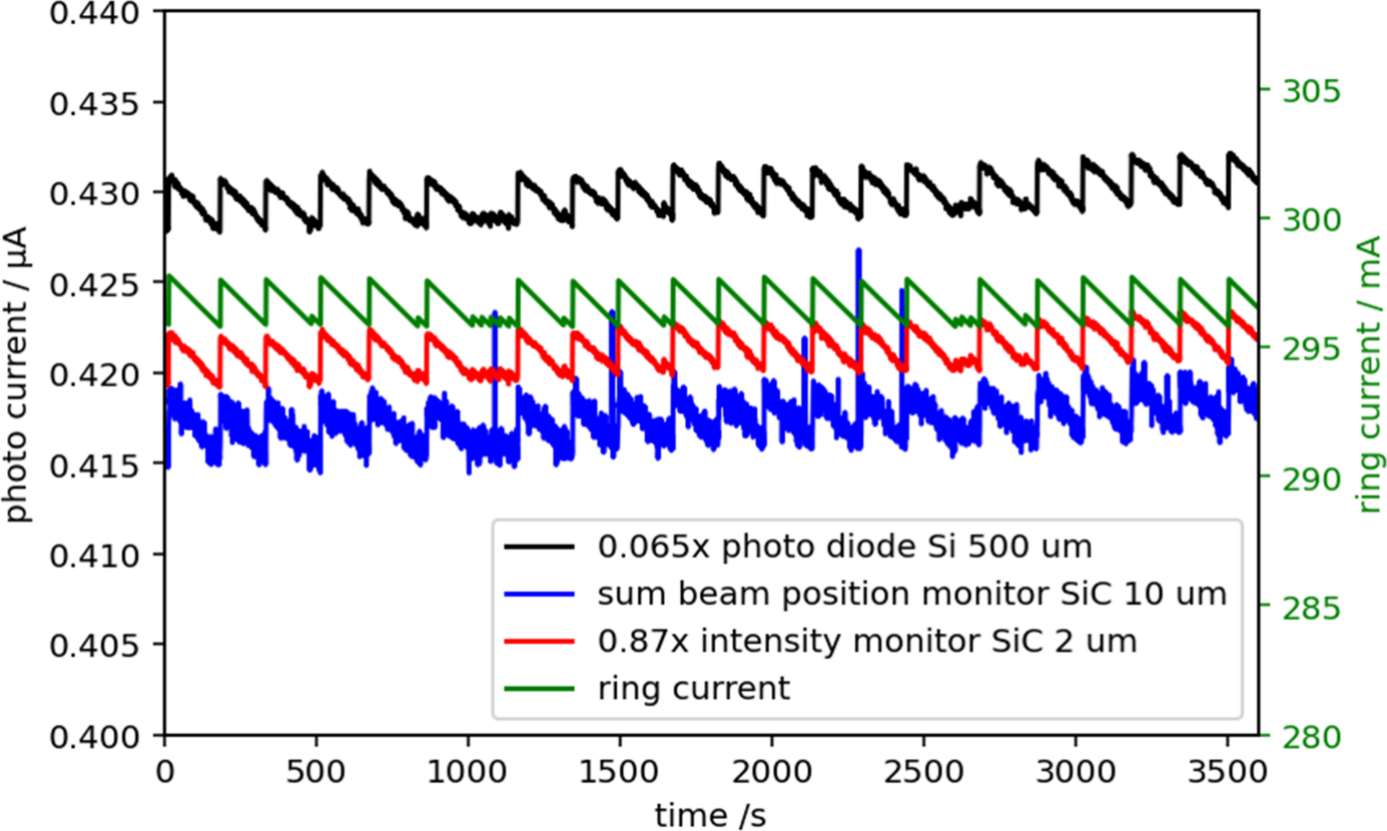
Test of the detector in XBIM mode using 10 keV X-rays with a photon rate of 1.5 × 10^10^ photons s^−1^. The sum current value is correlated with the ring current, including top-ups. Upon normalization, an RMS noise level of 0.1% is achieved.

Additional measurements aimed at characterizing the detector in the XBIM configuration were conducted using 10 keV X-rays with a photon flux of 1.5 × 10^10^ photons s^−1^ (Fig. 7 ▸). The sum of the signal collected by the four channels changes according to the time dependence of the beam intensity, including the top-up events, and coherently with the other two devices placed upstream and downstream to the rXBPM. The noise of the sum signal is, however, one order of magnitude larger than the noise measured by the beamline 2 µm SiC intensity monitor. After normalization, the noise is 0.1% RMS, although this value is still under investigation and should therefore be regarded as preliminary.

### 3.2. Characterization with the XOS laboratory source

**Figure 8:**
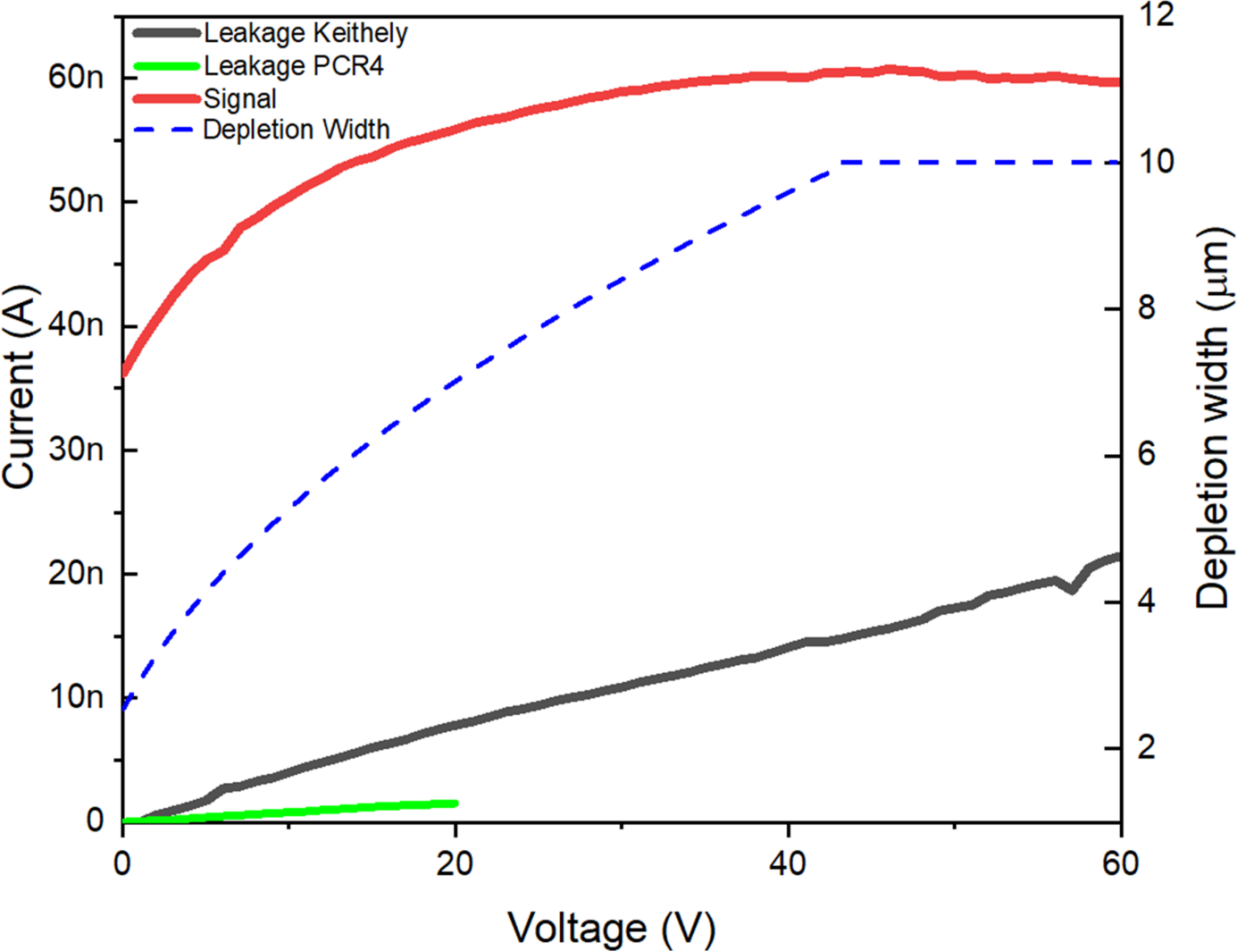
Current characteristics of the SenSiC rXBPM LEP polarized in reverse bias. The dark current of the diode is measured using the PCR4 picoammeter from 0 V to 20 V (green curve), and with the Keithley 2410 electrometer up to 60 V (black curve). The beam-induced current (red curve) is related to the theoretical depletion width (blue dashed curve) of the junction.

Fig. 8 ▸ shows the bias dependence of the beam-induced signal produced by the XOS X-ray tube used in the 30 kV and 1 mA configuration, the theoretical depletion width, and the leakage current measured using both a Keithley 2410 sourcemeter (Keithley Instruments, 1998 ▸) and a PCR4 picoammeter (SenSiC, 2024 ▸). The photoinduced current increases from about 36 nA at 0 V to approximately 60 nA and progressively approaches saturation for a bias voltage of 46 V. The increase of the collected charge closely follows the increase of the depletion width, which reaches the full active-layer thickness of 10 µm in the same voltage range. The leakage current remains much smaller than the signal when measured with the PCR4, compared with what is seen by the Keithley 2410. However, the PCR4 can only supply up to 20 V; for this reason, it was necessary to use a second instrument to explore the full collection of the detector. This measurement indicates a significant contribution from the measurement chain to the baseline current.

**Figure 9:**
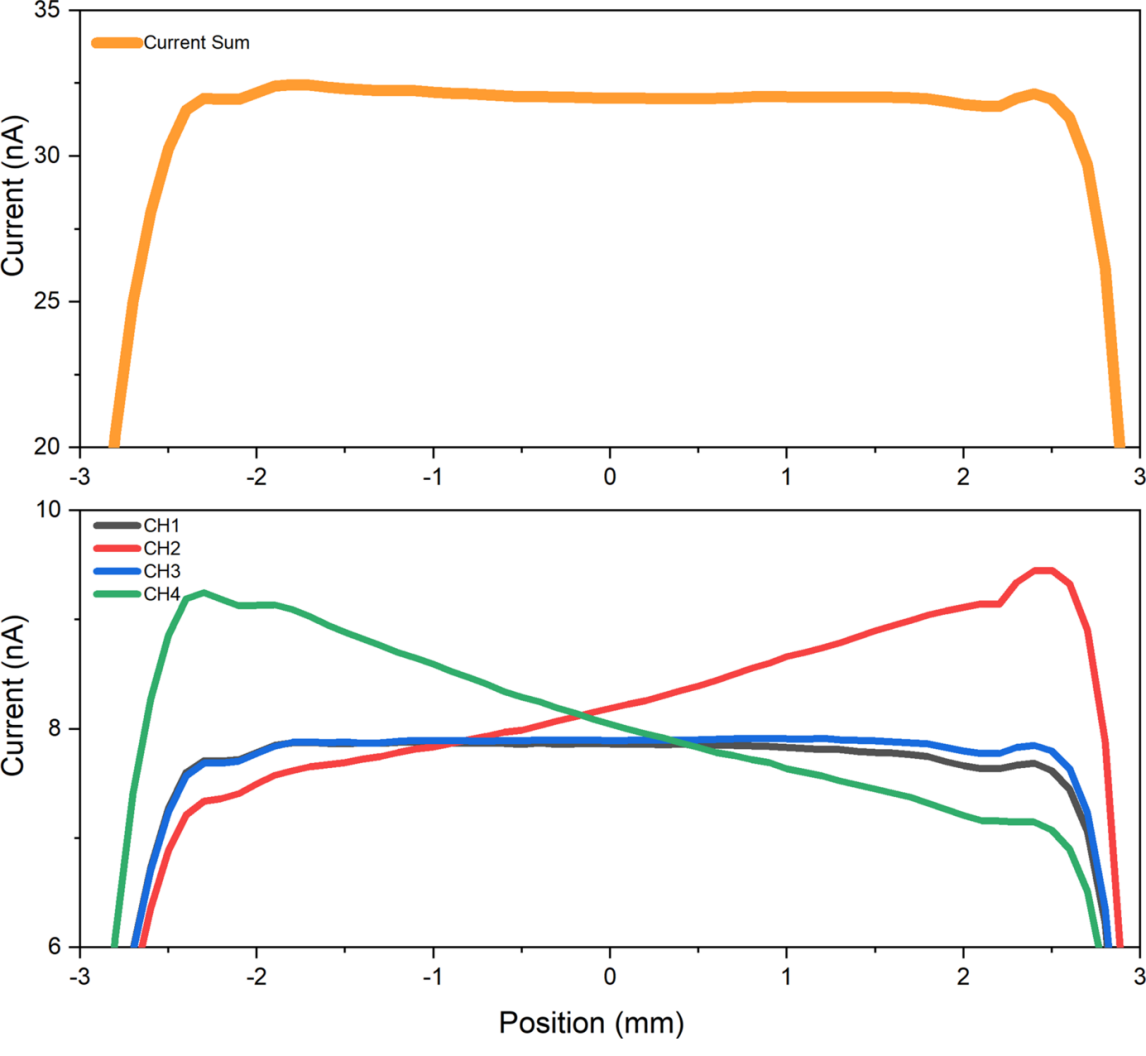
Line scan of the SiC rXBPM detector at 0 V bias, performed using the XOS X-ray laboratory source.

In Fig. 4 ▸(*b*) and Fig. 5 ▸, a current value lowering within the membrane in the −1 mm to 1 mm range is observed. The cause of this behavior is still under investigation. To exclude the possibility that this characteristic is related to the device configuration, a line scan spanning the *y*-axis in the position *x* = 0, using the second rXBPM, is presented in Fig. 9 ▸. The line scan is measured at 0 V using a PCR4 picoammeter to reproduce the same conditions of the previous measurement. The resulting line scan shows that no current dip in the region from −1 mm to 1 mm is present, confirming that the previously observed characteristic was related only to that sensor.

**Figure 10:**
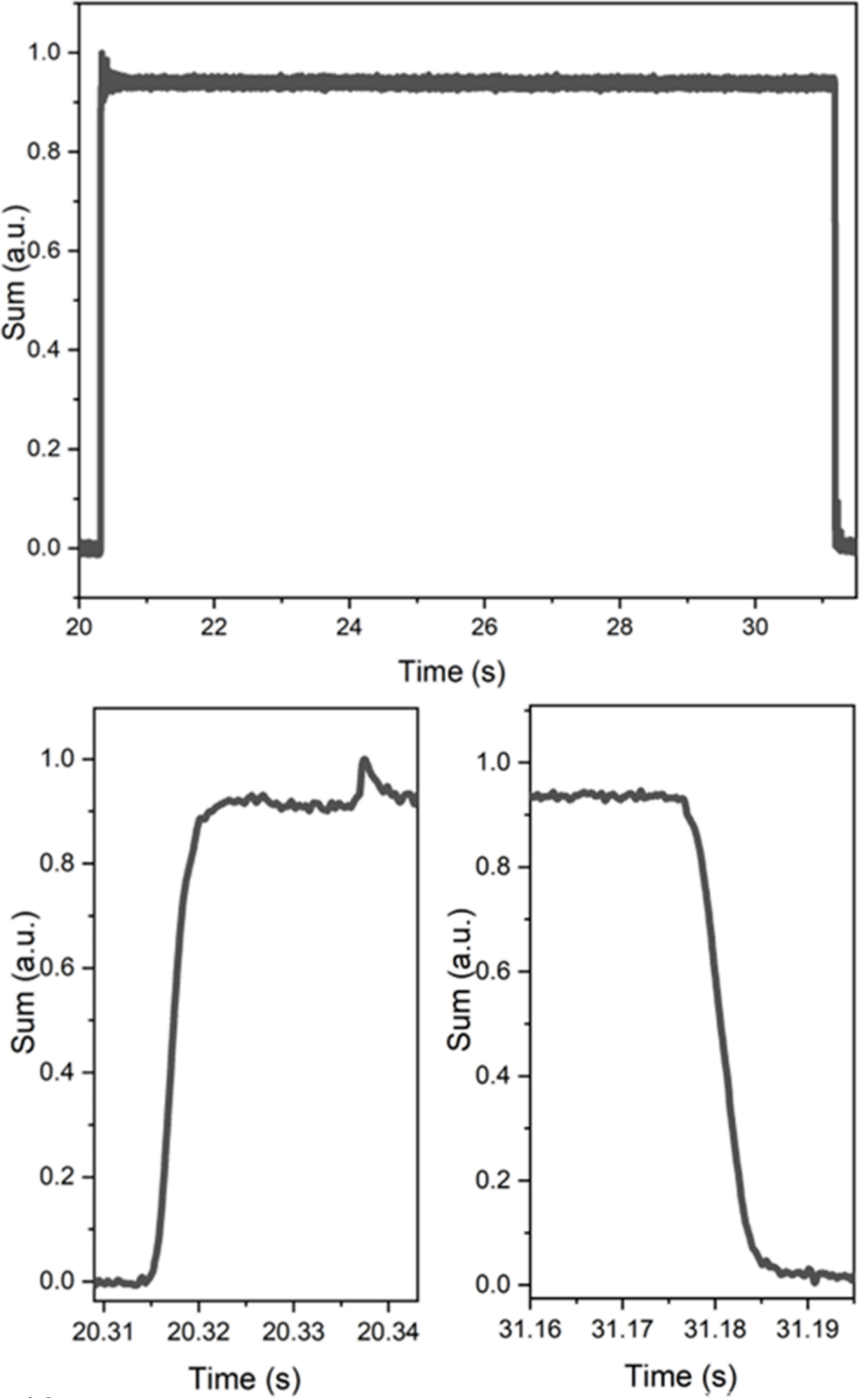
Temporal behavior of the SiC rXBPM recorded as the XOS X-ray tube shutter is opened and closed. The top graph is the normalized current sums from the four electrodes for the entire acquisition process, while the bottom graphs represent enlarged images of the rise and fall of the waveform, respectively. The rXBPM trace is directly tracking the light variation caused by the movement of the shutter on a millisecond timescale.

The temporal behavior of the rXBPM was observed by measuring the current sum recorded at the four electrodes with the XOS X-ray tube shutter opened and closed. The upper graph of Fig. 10 ▸ presents the whole recorded trace, while the magnification of the opening and closing processes is shown in the bottom graphs. The current quickly rises and decays back to its zero level. The switching occurs within a few milliseconds and it is governed by the shutter switching rather than the intrinsic characteristics of the SiC-based detector.

### 3.3. Simulation results

The simulated lateral response of a conventional XBPM and that of the resistive XBPM for different beam spot sizes, ranging from 50 µm to 2 mm FWHM, is represented in the supporting information. In the standard XBPM configuration, the photocurrent collected by each electrode is strongly influenced by the transverse size of the incident beam. Narrow beams generate strongly peaked current profiles centered around the electrode edges, while broader beams produce smoother and more extended distributions. As a result, the slope of the differential signals, *i.e.* the position sensitivity, strongly depends on the beam FWHM. This behavior is intrinsic to geometrical charge collection in segmented photodiodes: when the beam footprint varies, the fraction of charge generated within the depletion region close to each pad changes accordingly, which results in a nonlinear and non­uniform position response. By contrast, the rXBPM displays a remarkably different behavior: for all the studied beam sizes, the electrode currents vary linearly, with identical slopes. This stability reflects the action of the resistive charge-collection layer, which laterally redistributes the generated charge before it is collected by the electrodes. The discussed results are obtained through a two-dimensional simulation, which does not fully represent the charge transport properties of the detector.

**Figure 11:**
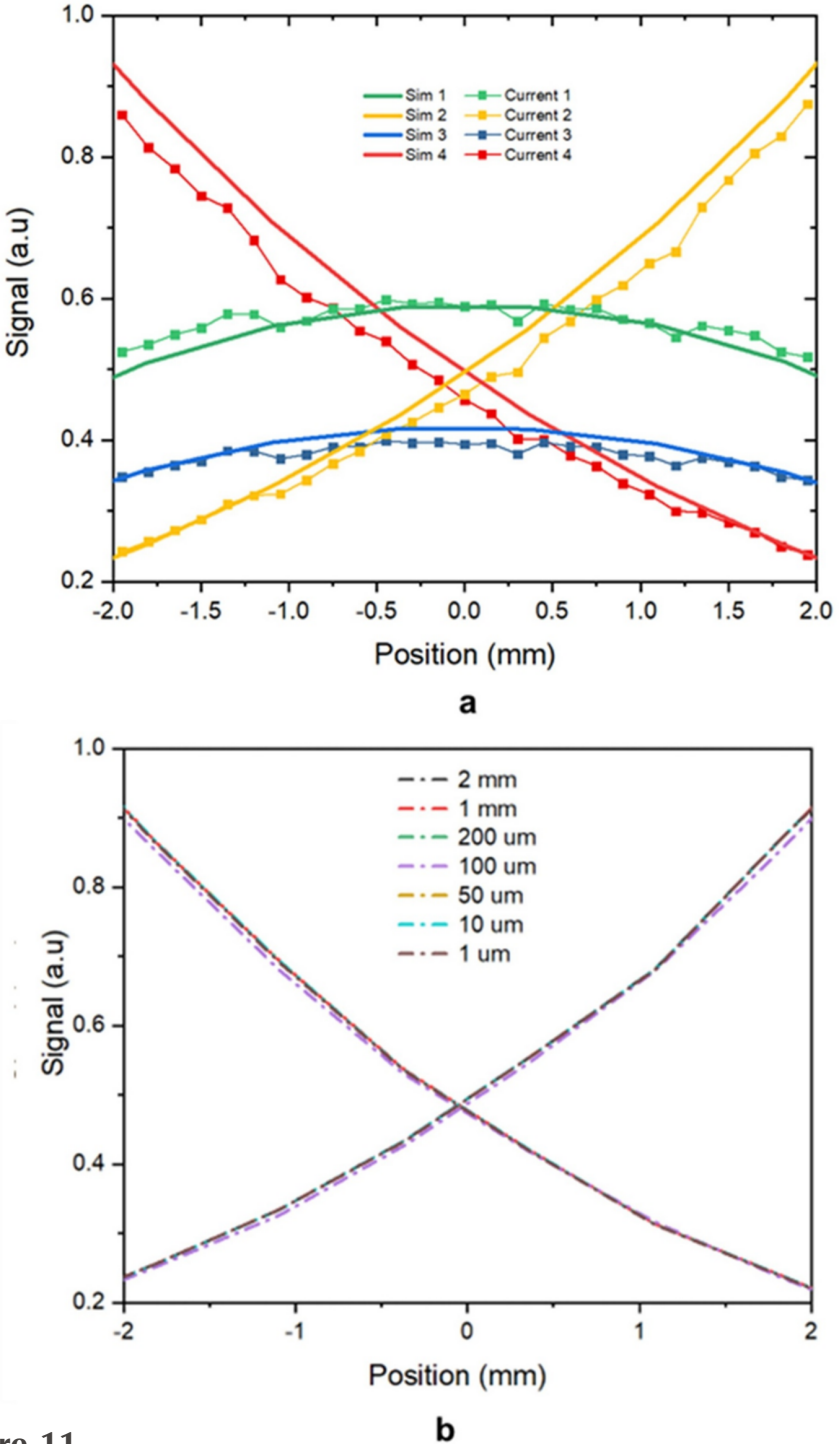
(*a*) Comparison between the experimental data (squares) and simulated results (solid lines) when irradiating the free-standing membrane. (*b*) Simulation repeated with different beam FWHMs, from 1 µm up to 2 mm.

To highlight the consistency of the simulated results, the three-dimensional simulation of the device was realized with the geometry and the material properties of the real detector. In Fig. 11 ▸(*a*) a comparison between the simulated results and the experimental points shown in Fig. 5 ▸ is presented. The simulation behaves just as the real device in the membrane area, having the typical lateral charge division of the tetralateral LEP devices.

In Fig. 11 ▸(*b*), a simulation of the response of the rXBPM to different FWHMs of the beam is represented. The FWHM is spanned from 1 µm up to 2 mm, and the simulation represents the behavior of the central area of the detector, *i.e.* where the free-standing membrane is located. An immediate consequence is that the rXBPM position sensitivity is independent of the beam FWHM; therefore, it operates effectively over a very wide range of beam conditions without the necessity for device-specific calibration as a function of each specific beam size.

Although the simulation successfully reproduces both the current crossover and the characteristic bowing of the *Y*-response, some deviations are evident at larger distances from the center. These differences are attributed to the simplified model, which considers idealized electrode representation and does not take into account uncertainties in the effective conductivity of the resistive layer, membrane non-uniformity, and small experimental alignment errors.

## 4. Conclusions

In this study, a silicon carbide resistive X-ray beam position monitor based on the lateral effect principle has been designed, developed, and experimentally validated. The sensor uses a resistive p^+^-doped SiC thin layer and a free-standing membrane structure created by a selective electrochemical etching process, enabling intensity and position monitoring in transmission mode, making the device suitable as synchrotron beam diagnostics. Experimental characterization at the MiFo beamline of the BESSY II synchrotron demonstrated an average X-ray transmission of approximately 61% at 5.4 keV with good spatial homogeneity. A quantitative position sensitivity of ≥0.157 mm^−1^ and estimated upper-limit noise-equivalent positions of 2.7 µm and 4.7 µm were obtained for the ±500 µm and ±1 mm ranges, respectively, confirming the suitability of the device for precise and non-invasive beam diagnostics applications and beam position monitoring.

Experimental results, supported by 3D COMSOL simulations, demonstrate that the position sensitivity of the resistive XBPM is independent of the beam spot size, unlike conventional XBPMs. This characteristic makes the resistive XBPM very useful due to its independence of beam size, making this a proof-of-principle beam spot size calibration-free, compact, and radiation-resistant candidate for X-ray beam position monitoring.

Beyond vacuum operation at synchrotron facilities, rXBPMs with different active layer thicknesses were also validated in air using a focused and monochromated laboratory X-ray source, demonstrating the robustness and versatility of the detector architecture. SiC devices can, in principle, be fabricated with significantly larger active areas than diamond-based position-sensitive detectors, potentially reaching the several cm^2^ scale. This size is beneficial to those beamlines with alignment constraints and enables beam monitoring even in configurations where precise positioning is not easily obtainable. The membrane-based SiC structure has already been proven to go below 1 µm in thickness, enabling tender X-ray beamlines, where minimal absorption and highly transparent diagnostics are essential, to implement this diagnostic element. This study confirms SiC rXBPMs as a new promising solution for beam position control, offering promising prospects for any future improved ultra-thin, large-area versions for covering the needs of synchrotron radiation sources.

## Supplementary Material

Figs. S1, S2 and S3. DOI: 10.1107/S1600577526005242/ys5111sup1.pdf
