# Supplementary material for: SiC resistive X-ray beam monitor for intensity and position control of synchrotron light
Source: J Synchrotron Radiat. 2026 Jun 18;33(Pt 4):995–1004. doi: 10.1107/S1600577526005242 (PMC13344595; doi:10.1107/S1600577526005242)
Supplement: Supplementary file 1 [file s-33-00995-sup1.pdf]

JOURNAL OF SYNCHROTRON RADIATION

Volume 33 (2026)

Supporting information for article:

SiC resistive X-ray beam monitor for intensity and position control of synchrotron light

Gabriele Trovato, Niccolò La Rosa, Francesco La Via, Janin Lubeck, Antonio Manno, Samuele Moscato, Matthias Müller and Massimo Camarda

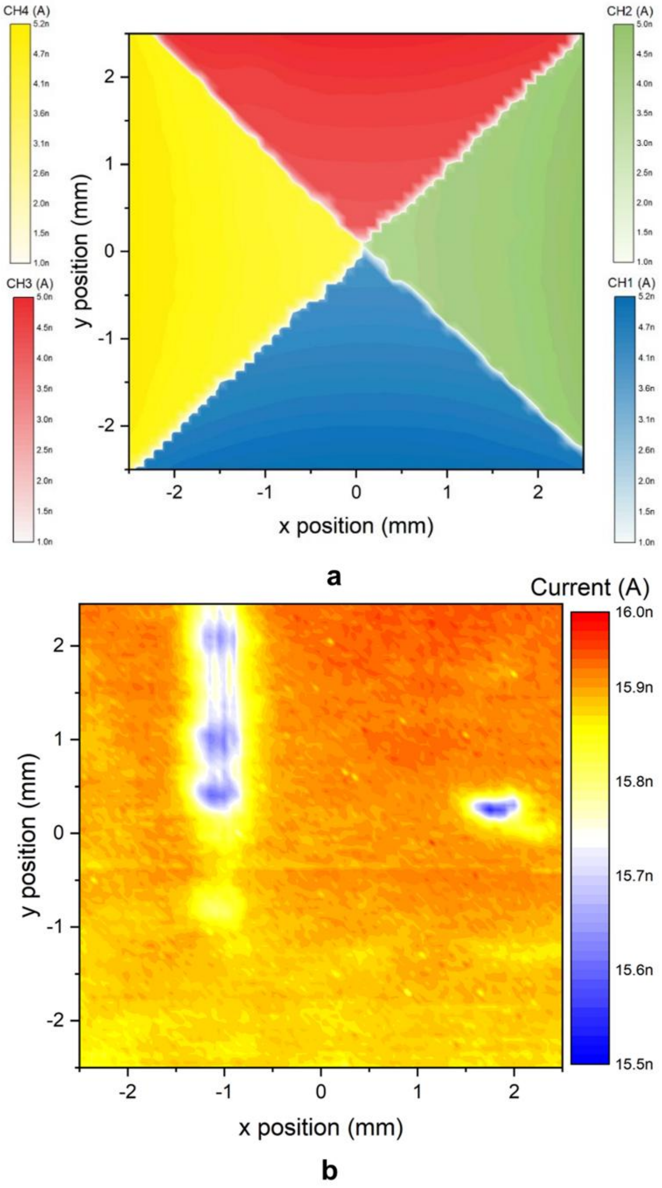

Figure S1 Photoinduced current map on the 34 μm thick bulk SiC LEP detector tested with the XOS 5.4 KeV X-ray tube (XOS, 2026). (a) Currents collected by the four channels and (b) sum of the four currents.

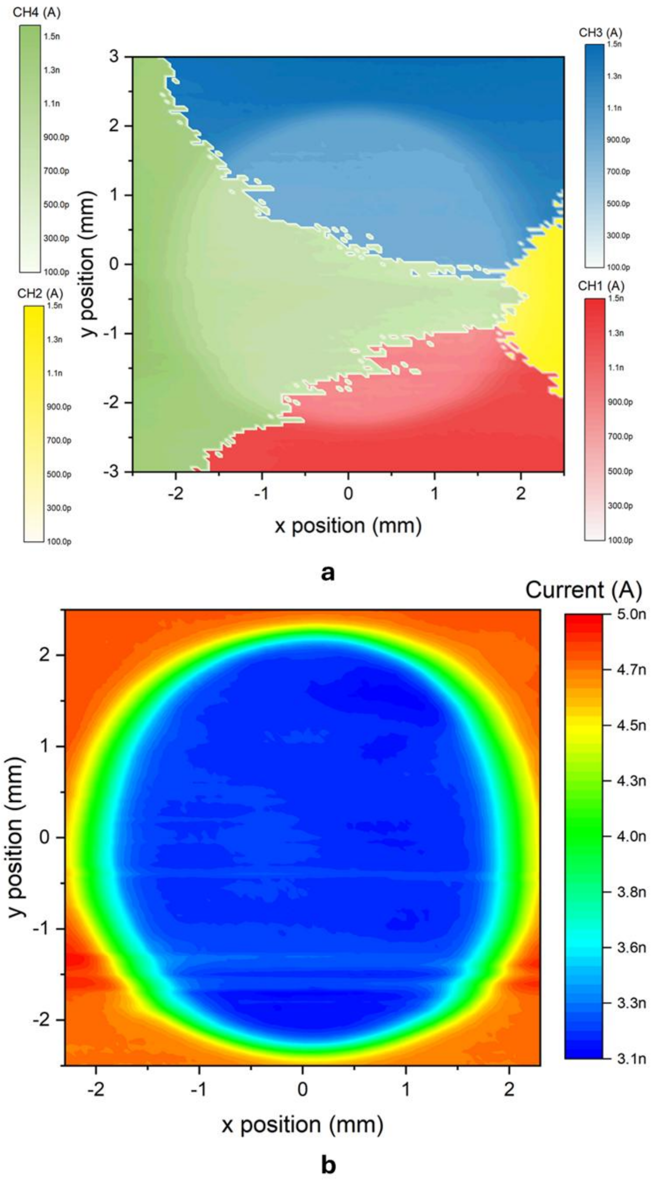

Figure S2 Photoinduced current map on the 1 μm thin membrane SiC LEP detector tested with the XOS 5.4 keV X-ray tube (XOS, 2026). (a) Currents collected by the four channels and (b) sum of the four currents. The lighter area suggest the lower current production in the membrane area with respect to the bulk region.

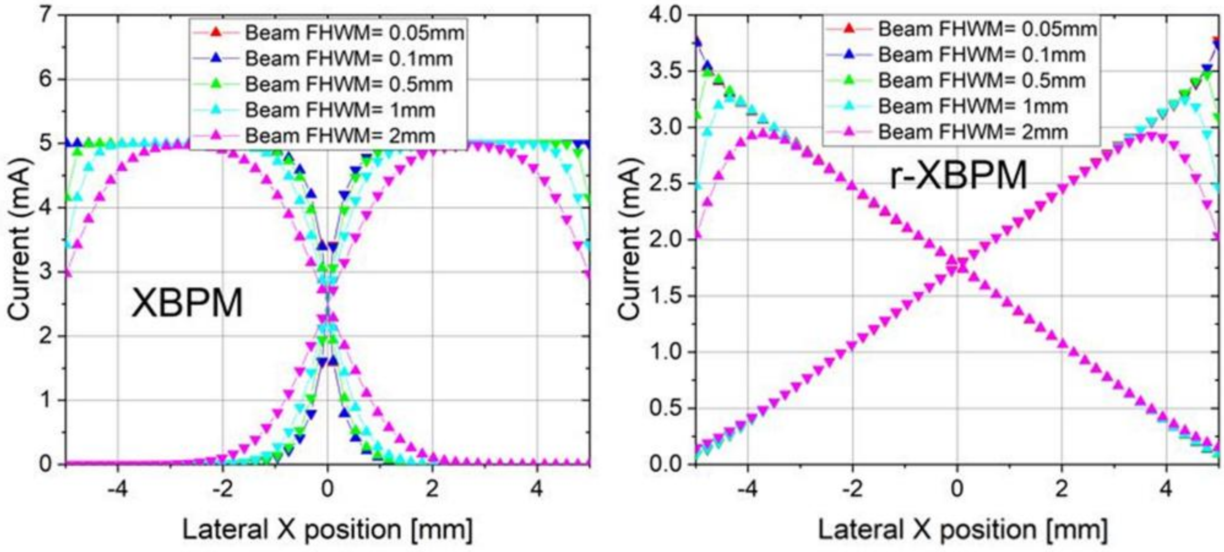

Figure S3 Simulation of the response of a standard XBPM (left) and a rXBPM (right) when varying the FWHM of the beam scanning the device on the x axis.
